# Imaging lung manifestations of HIV/AIDS

**DOI:** 10.4103/1817-1737.69106

**Published:** 2010

**Authors:** Carolyn M. Allen, Hamdan H. AL-Jahdali, Klaus L. Irion, Sarah Al Ghanem, Alaa Gouda, Ali Nawaz Khan

**Affiliations:** *North Manchester General Hospital, Pennine Acute NHS Trust, Manchester, UK*; 1 *King Fahad Hospital King Abdulaziz Medical City, Riyadh, Saudi Arabia*; 2 *The Cardiothoracic Centre Liverpool NHS Trust, The Royal Liverpool University Hospital, UK*

**Keywords:** HIV/AIDS, imaging lung, mediastinal manifestations

## Abstract

Advances in our understanding of human immunodeficiency virus (HIV) infection have led to improved care and incremental increases in survival. However, the pulmonary manifestations of HIV/acquired immunodeficiency syndrome (AIDS) remain a major cause of morbidity and mortality. Respiratory complaints are not infrequent in patients who are HIV positive. The great majority of lung complications of HIV/AIDS are of infectious etiology but neoplasm, interstitial pneumonias, Kaposi sarcoma and lymphomas add significantly to patient morbidity and mortality. Imaging plays a vital role in the diagnosis and management of lung of complications associated with HIV. Accurate diagnosis is based on an understanding of the pathogenesis of the processes involved and their imaging findings. Imaging also plays an important role in selection of the most appropriate site for tissue sampling, staging of disease and follow-ups. We present images of lung manifestations of HIV/AIDS, describing the salient features and the differential diagnosis.

Human immunodeficiency virus (HIV)/acquired immunodeficiency syndrome (AIDS) remains a critical world health issue and is a major cause of morbidity and mortality. More than 25 million people have died of AIDS since 1981. Africa has 11.6 million AIDS orphans.[1]

The lungs are one of the chief target organs for HIV-associated disease, and almost 70% of the patients suffer at least one respiratory complication during the course of their illness.[2–4] In the HIV patient, *there is an enhanced* prevalence of bronchial hyperresponsiveness and dysfunction of the small airways.[5,6]

Recently gained knowledge of the immunologic impact of HIV on disease progression has led to better care and incremental increase in survival. Better prophylaxis for opportunistic infections and the development of highly active antiretroviral therapy (HAART) has had a significant impact against HIV. HAART has had a significant impact on viral load, CD^4+^ cell count and HIV-related mortality both in adults[7] and in pediatric populations[8] (75% and 67% reductions, respectively, in the risk of death with HAART).[8] Similar reductions in the incidence of both community-acquired pneumonia[9] and opportunistic infections, including *Mycobacterium tuberculosis (MTB)*, cytomegalovirus (CMV) and *Pneumocystis carinii* pneumonia (PCP), have been recently reported with HAART.[2,7,10,11]

Coupled with the wide spectrum of pulmonary diseases encountered in AIDS summarized in Table 1, this poses a considerable challenge for the radiologist and the attending physician. It is therefore imperative that we use an integrated approach in the interpretation of imaging. Pattern recognition should be combined with knowledge of clinical factors in order to generate a limited and meaningful differential diagnosis [Table 2]. For example, Kaposi sarcoma (KS) is seen almost exclusively in homosexual and bisexual men and their partners[12–14] whereas IV drug abusers are particularly prone to bacterial infections and MTB.[15,16]

**Table 1:** Pulmonary manifestation of HIV / AIDS

Opportunistic infection
Drugs reaction
Immune restoration syndrome
Lymphoproliferative disorders
AIDS related malignancy
Non-specific interstitial pneumonitis
HIV related pulmonary hypertension
Bronchiolitis obliterans
Emphysema and bronchiectasis

**Table 2:** Intergation of clinical date

Based of CD4 CD >400 : Increase risk of Bactetial infection Mycobacterium tuberculosis CD4 200-400 : Increase risk for Recurrent bacterial infections Mycobacterium tuberculosis Lymphoma and Lymphoproliferative disorders CD4 <200 : Increase risk for PCP Disseminated *Mycobacterium tuberculosis* CD4 <100 : Increase risk of PCP A typical *Mycobacterium tuberculosis* CMV Kaposi’s sarcoma Lymphoma Others Transmission rout: Kaposi’s sarcoma is almost exclusively in homosexual and bisexual men and their partners Intravenous drugs abuser: at increase risk of infection and *Mycobacterium tuberculosis* Being on therapy, Steroid, HAART, prophylaxes therapy Prior history of infection Overall patients status and clinical presentation

A productive cough is distinctly atypical for PCP and should suggest pyogenic infection, whereas miliary nodularity may reflect disseminated fungal infection or tuberculosis (TB) in a sick patient. In a relatively well patient, it likely represents a more indolent process such as lymphoproliferative disorder.[17–23]

Steroids may increase the degree of immune suppression and HAART can lead to various manifestations of immune restoration syndrome.[24] A history of previous opportunistic infection has been shown to increase the risk of a further episode, even when other factors such as the CD4 level are taken into account.[25]

However, the level of immune compromise is the most important determinant in assessing the likelihood of various pulmonary complications. This is reflected in the CD4 count, with specific diseases occurring with increasing frequency as certain CD4 thresholds are reached. At CD4 levels above 400, patients are at risk of infection from relatively virulent organisms, such as bacteria and TB. Lung cancer may also occur at this stage. Between counts of 200 and 400, patients may suffer from recurrent infection as well as lymphoma. Opportunistic infections and KS are rare at CD4 levels >200 and indeed most cases of PCP occur at CD4 counts below 100, along with *Mycobacterium avium* complex (MAC), fungal infections and CMV[26] [Table 2].

The conventional chest X-ray (CXR) is usually the first line and often only imaging investigation. Despite the variety of differential diagnoses, overlapping features, atypical manifestations and multifactorial disease, the CXR is accurate for diagnosing common complications, with reported accuracies of 64%, 75% and 84% for diagnosing bacterial pneumonia, PCP and MTB, respectively, in a blinded trial.[27]

High-resolution computed tomography (HRCT) is both more sensitive and more accurate, with an overall diagnostic accuracy of 92%, including 90% for KS and 94% for PCP. Furthermore, CT has a useful 93% negative predictive value in excluding active pulmonary disease.

When comparing CXR and HRCT, Kang found that a confident diagnosis was made more often on CT than on CXR, and this confident diagnosis was more often correct on CT than on CXR. The correct confident interpretation was made more often in patients with lymphoma (100%), KS (91%) and PCP (87%).[28] However, CT only affords a modest improvement and is therefore not routinely indicated[29] [Table 3]. Specific indications include the detection of occult disease in symptomatic patients, characterizing nonspecific radiographic disease patterns, assessment of the mediastinum, staging malignancy, assessing complications (particularly pleural disease) and guiding invasive procedures.

**Table 3:** Indication for CT / HR CT

Characterizing non specific CXR abnormalities Detection of occult lung disease Evaluation of the mediastinum Staging of malignancy Assessing complications Guiding interventional procedures

## Clinical and Radiologic Manifestation

Clinical findings vary with the degree of immune suppression, ranging from an increased incidence of bronchitis and sinusitis, with CD4 counts above 500/µL, to PCP and KS, with CD4 counts below 200/µL.[4] The introduction of HAART has succeeded in considerable reduction in mortality from AIDS in both adult and pediatric populations.[7,8] A similar reduction has been achieved in the incidence of both community and acquired pneumonia.[2,7,9–11]

## Bacterial Infection

Infections are the most common pulmonary complication of AIDS, and bacteria are the most common cause of infection, superseding PCP at all levels of immune compromise.

Multiorganism infection is common, involving *Streptococcus pneumoniae, Hemophilus influenzae,* Pseudomonas and *Staphylococcus aureus*. Infection is frequently not pneumonic, the most common manifestation being acute bronchitis/bronchiolitis.[30–32] Patients with bacterial pneumonia typically present with an acute onset of fever and productive cough.[33]

**Figure 1:**
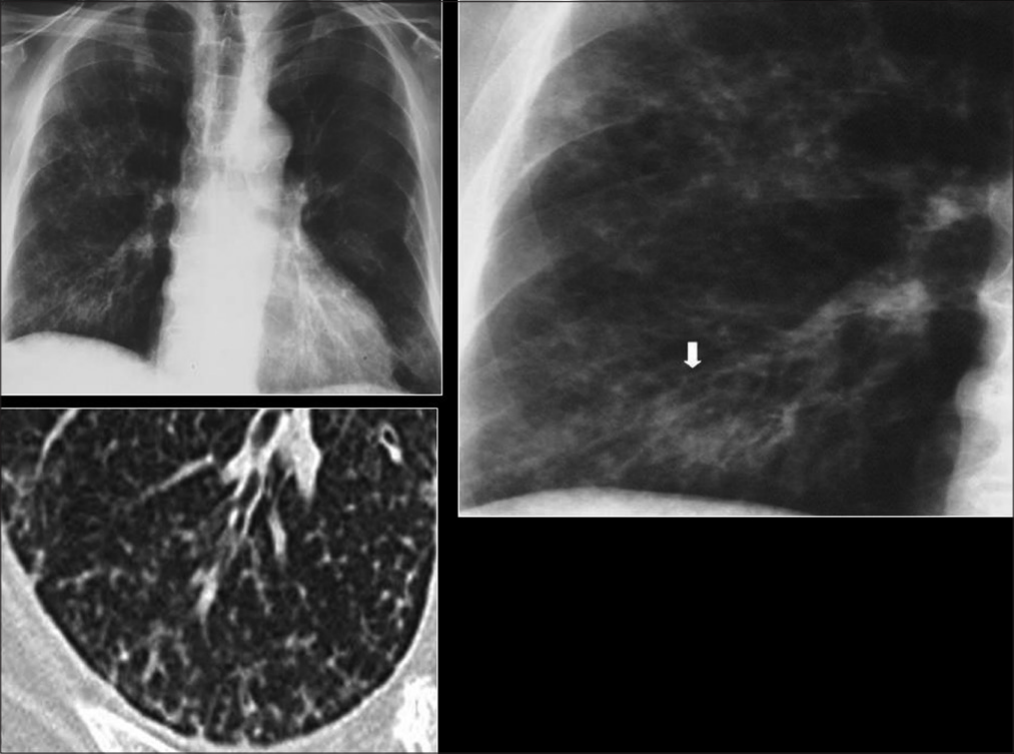
Bacterial bronchiolitis. Chest X-ray of a 36-year-old human immunodeficiency virus-positive male with a CD4 count of >400 presenting with productive cough. Note (left) the peribronchial thickening. The right hand film is a blown-up image showing the peribronchial thickening more clearly (arrow). The high-resolution computed tomography (lower left) depicts bronchiectasis, centrilobular nodularity/“tree-in-bud” mosaic attenuation in the same patient

The CXR in bacterial bronchiolitis may be normal or may reveal bilateral symmetrical lower lobar bronchial wall thickening appearing as ring shadows and “tram-tracks”[34] [Figure 1]. Abnormalities may be detected on HRCT in the absence of any CXR findings [Figure 1]. These include bronchiectasis and evidence of small airway disease, with ill-defined centrilobular micronodularity and branching structures or tree-in-bud appearance secondary to mucus impaction in the bronchioles. Mosaic attenuation may also be present due to air trapping.[34]

**Figure 2:**
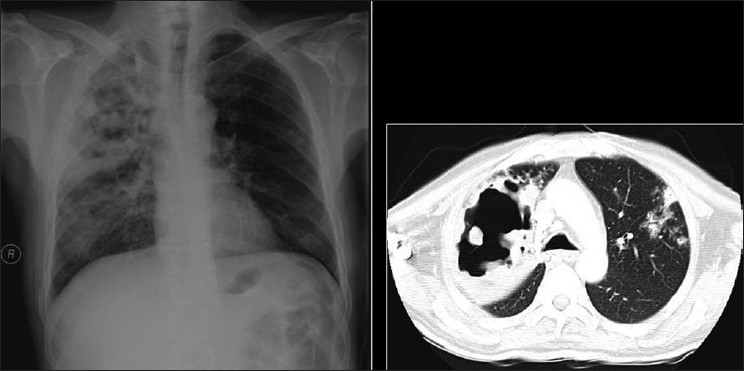
Necrotizing cavitating pneumonia. Chest X-ray and computed tomography depicting necrotizing cavitating pneumonia due to *Staphylococcus aureus* in a 29-year-old male with acquired immunodeficiency syndrome

**Figure 3:**
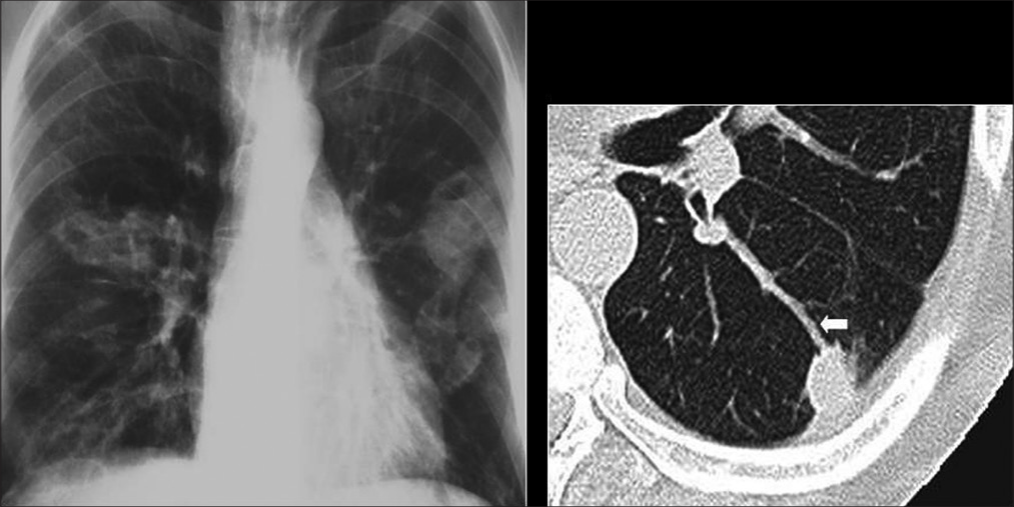
Septic emboli. A chest X-ray (CXR) and high-resolution computed tomography (HRCT) on a human immunodeficiency virus-positive patient with gram negative septicemia and septic emboli. Note the wedge-shaped pleural-based opacity and the feeding vessel on HRCT (arrow) and multiple cavitating nodules on the CXR

**Figure 4:**
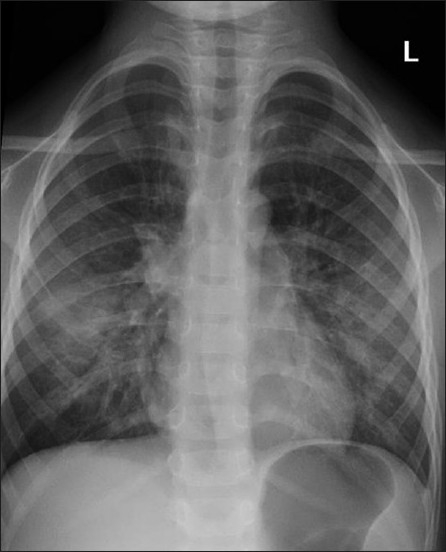
A chest X-ray of a patient with a CD4 count <200/mm^3^ showing perihilar ground-glass appearance in the shape of bats-wings

The typical radiographic appearance of bacterial pneumonia CXR in AIDS is the same as that in an immune competent host with lobar or segmental consolidation but progressing rapidly, with frequent multilobar or bilateral disease[27,35,36] [Figure 2]. Parapneumonic effusions and empyema are common. Cavitation may occur,[35,36] particularly with septic emboli and gram-negative infection [Figures 3 and 4]. A useful ancillary finding is the identification of a characteristic feeding vessel (arrow) on CT [Figure 4]. Although pyogenic infections are the most common cause of these imaging findings in AIDS patients, viral and mycobacterial infections may produce similar appearances.[34]

## PCP

**Figure 5:**
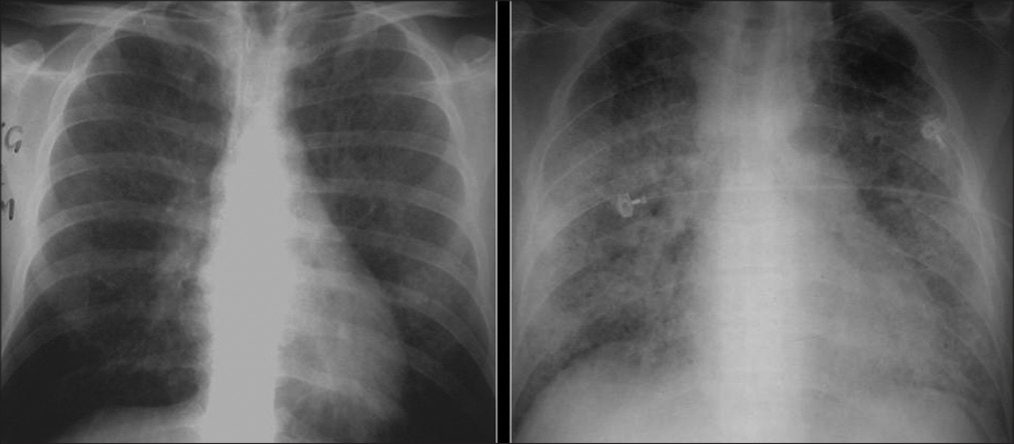
*Pneumocystis carinii* pneumonia. These chest radiographs are of two patients. Both show -ground glass appearance. The left chest X-ray (CXR) shows a much more subtle ground-glass appearance while the right CXR shows a much more gross ground-glass appearance mimicking pulmonary edema

**Figure 6:**
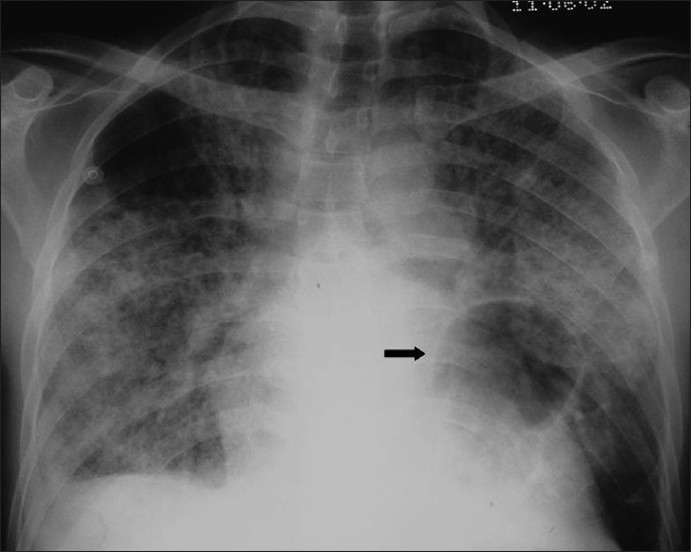
*Pneumocystis carinii* pneumonia. If left untreated, chest X-ray may progress to alveolar consolidation in 3 or 4 days. Infiltrates clear within 2 weeks, but in a proportion infection will be followed by coarse reticular opacification and fibrosis. Note the large cyst (arrow)

**Figure 7:**
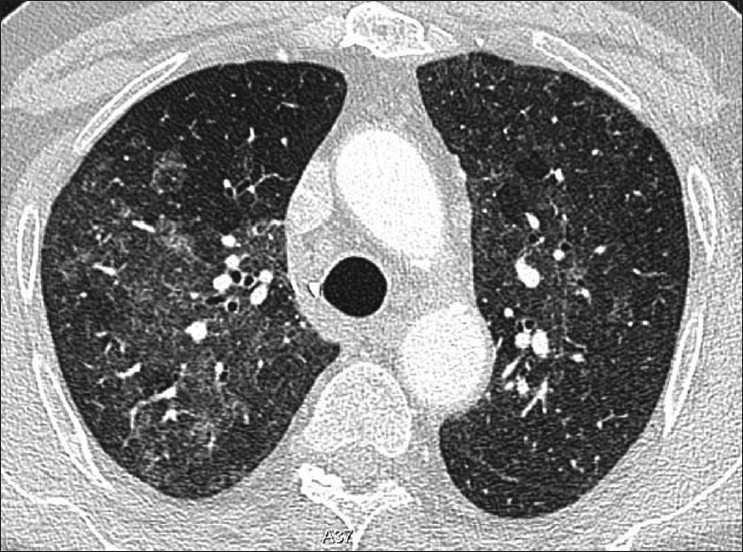
*Pneumocystis carinii* pneumonia. Computed tomography (CT) in a subacute phase showing foci of consolidation and interlobular septal thickening due to organized inflammatory infiltrate on high-resolution CT

**Figure 8:**
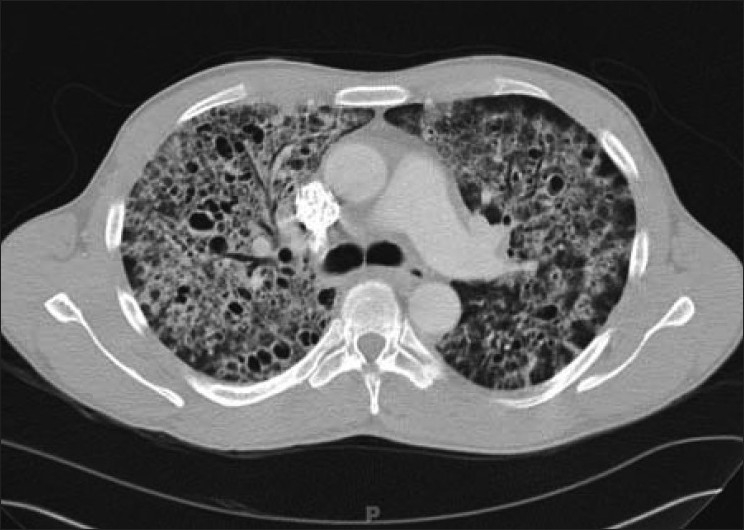
*Pneumocystis carinii* pneumonia (PCP). High-resolution computed tomography showing the hallmark of PCP in a clinical setting of immune compromise. Note the ground-glass attenuation with a geographic or mosaic distribution

**Figure 9:**
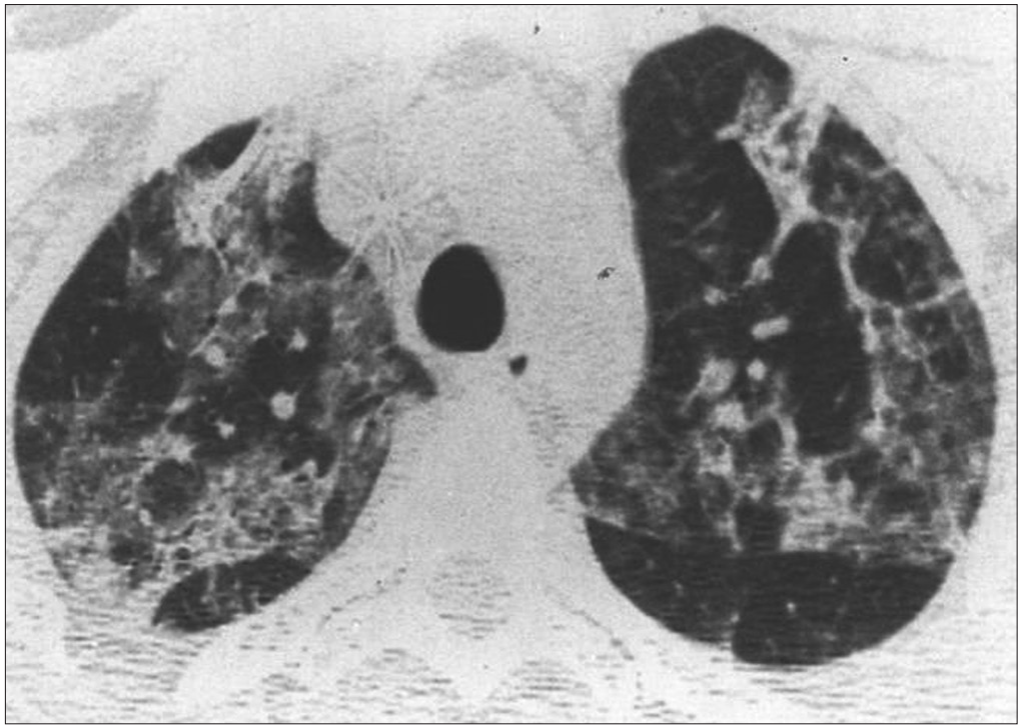
*Pneumocystis carinii* pneumonia. Computed tomography (CT) in a subacute phase showing foci of consolidation and interlobular septal thickening due to organized inflammatory infiltrate on high-resolution CT

*P. carinii* remains the most common opportunistic and most common life-threatening pulmonary infection in AIDS.[37–39] PCP usually occurs in patients not receiving medical care.[40,41] Radiographic changes are varied and may lag behind the symptoms.[40] The classic appearance is of bilateral symmetric perihilar or diffuse interstitial opacification, which may be reticular, finely granular or ground-glass in appearance[40] [Figures 5–7]. If left untreated, this may progress to alveolar consolidation in 3 or 4 days. Infiltrates clear within 2 weeks but in a proportion, infection will be followed by coarse reticular opacification and fibrosis[40] [Figures 8 and 9].

**Figure 10:**
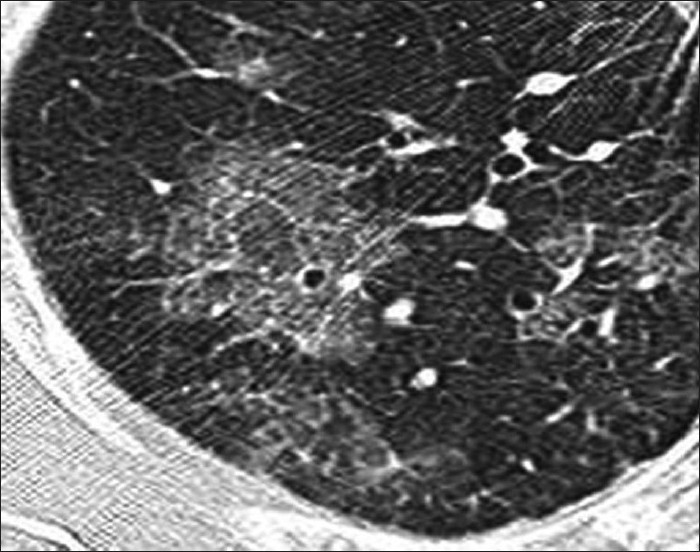
*Pneumocystis carinii* pneumonia (PCP). High-resolution computed tomography showing the hallmark of PCP in a clinical setting of immune compromise. Note the ground-glass attenuation with a geographic or mosaic distribution

**Figure 11:**
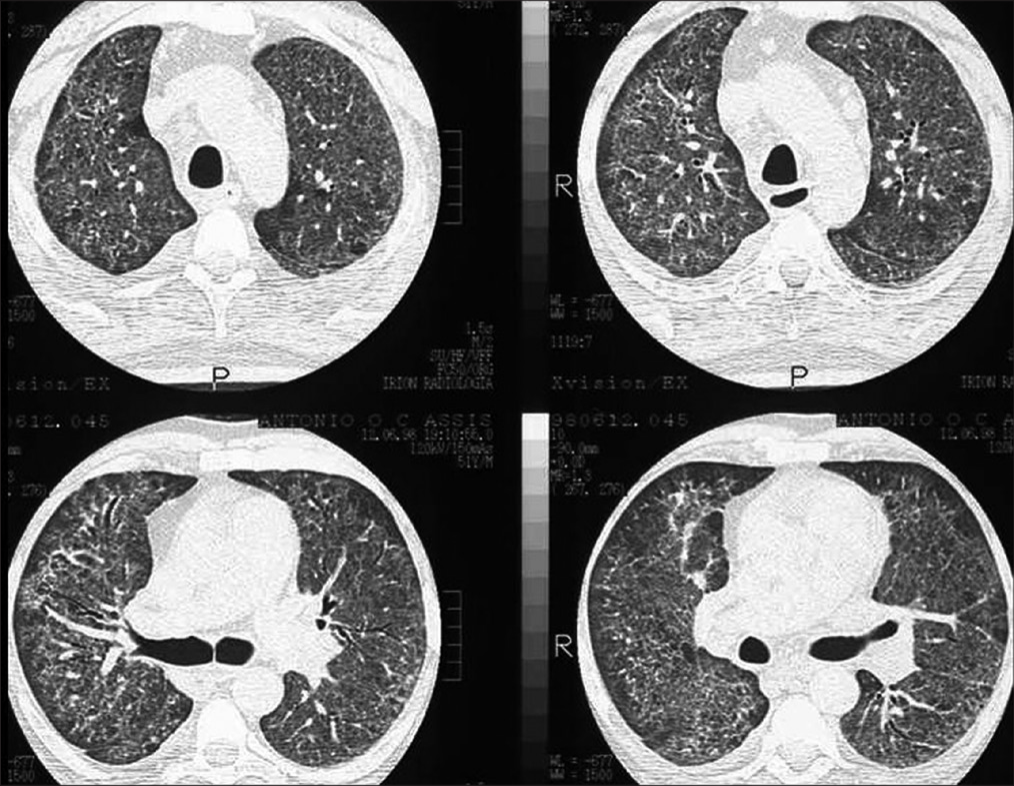
*Pneumocystis carinii* pneumonia. High-resolution computed tomography shows diffuse ground-glass attenuation with a geographic distribution

HRCT is more sensitive, the hallmark being ground-glass attenuation, seen in over 90% of the cases. It often has a geographic or mosaic distribution. In this clinical setting, this is virtually diagnostic of PCP and is sufficient for commencement of empirical treatment[40] [Figures 10 and 11]. Foci of consolidation and interlobular septal thickening may be seen in the subacute stage due to organized inflammatory infiltrate[40] [Figures 10 and 11].

**Figure 12:**
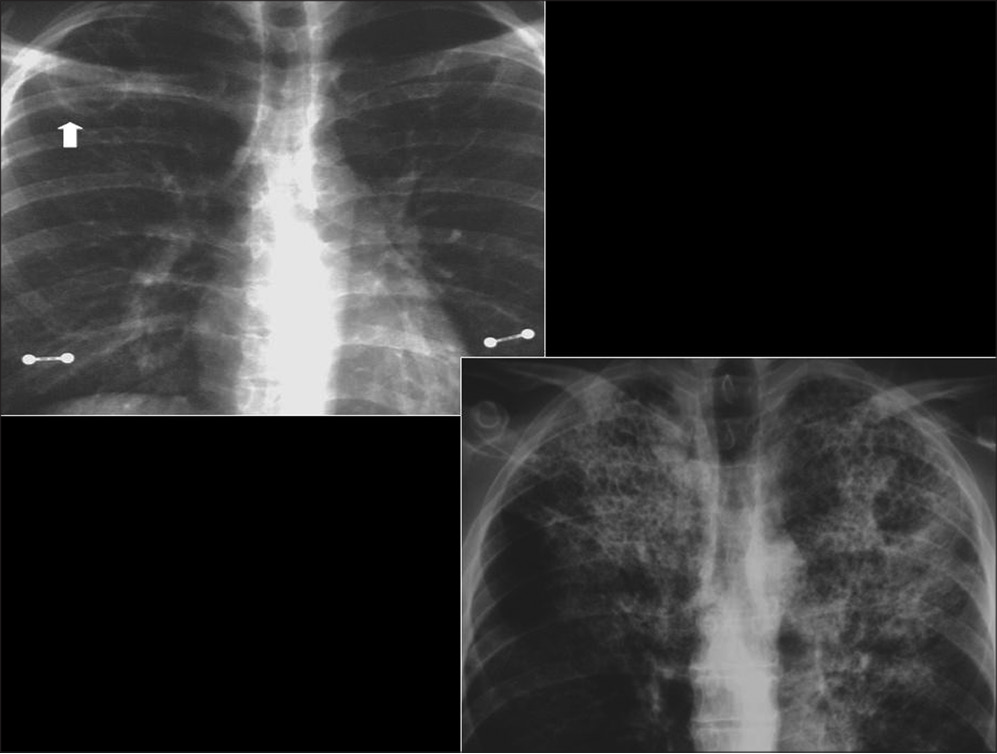
*Pneumocystis carinii* pneumonia. Chest X-ray (R) shows a thin-walled cyst in the right upper lobe (arrow). The left image shows multiple cysts in the apical regions related to pentamidine inhalation in a human immunodeficiency virus patient

**Figure 13:**
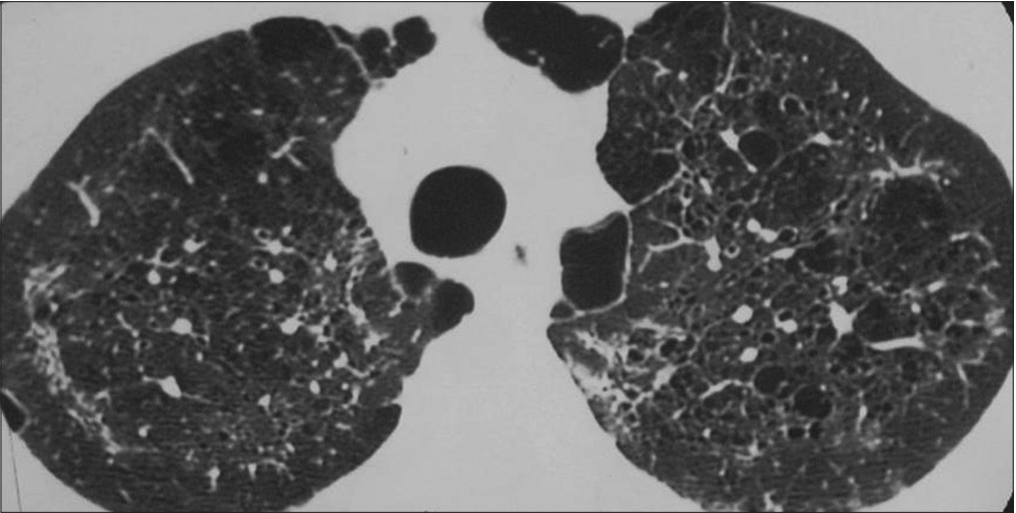
*Pneumocystis carinii* pneumonia. Lung cysts are usually multiple and bilateral, but range in size, shape and distribution. They are more commonly appreciated on computed tomography (CT)/high-resolution CT

Cystic lung disease is a relatively common manifestation.[35,40] Cysts are usually multiple and bilateral, but range in size, shape and distribution[35,40] [Figure 12]. They may develop in the acute or postinfective period and have been reported to remain for up to 3 years. They are more commonly appreciated on CT/HRCT, being reported in up to one-third of the patients [Figure 13].

**Figure 14:**
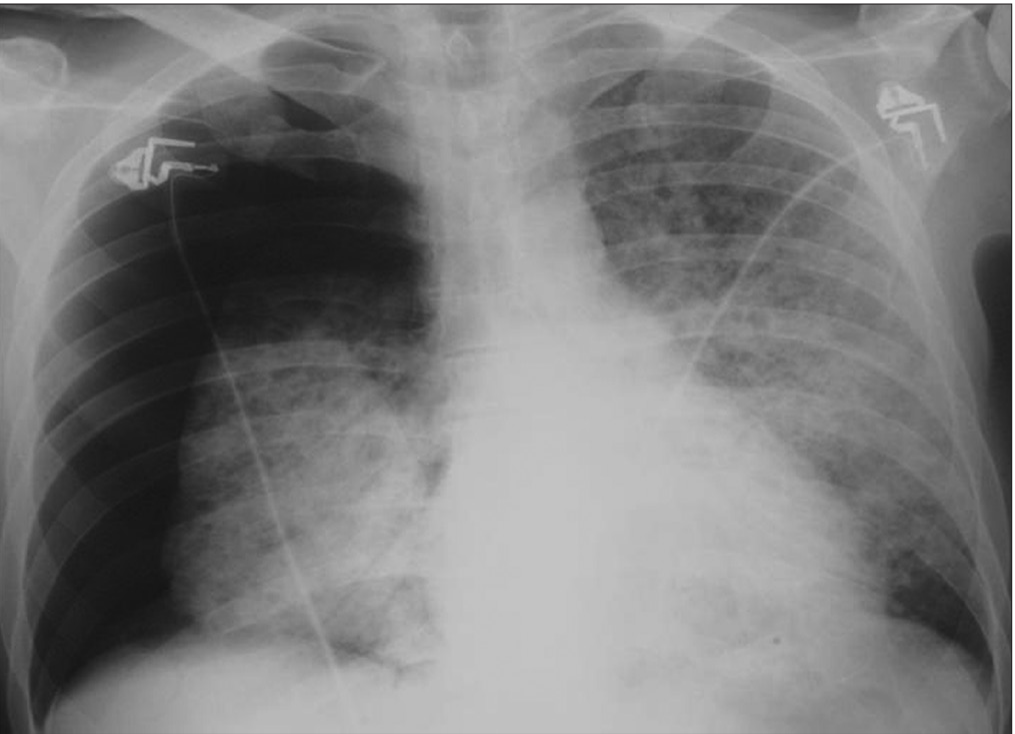
*Pneumocystis carinii* pneumonia. Chest X-ray showing an approximately 50% right-sided spontaneous pneumothorax

**Figure 15:**
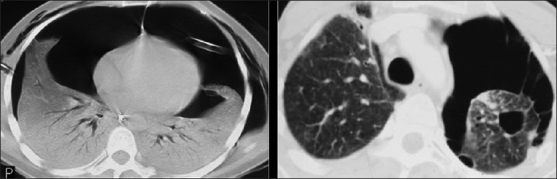
*Pneumocystis carinii* pneumonia. Computed tomography showing bilateral pneumothoraces in conjunction with lung cysts

**Figure 16:**
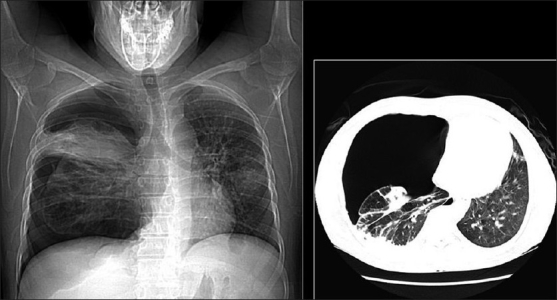
*Pneumocystis carinii* pneumonia. Chest X-ray and computed tomography show a left-sided ground-glass pattern and a right-sided large tension pneumothorax. Note the mediastinal shift

**Figure 17:**
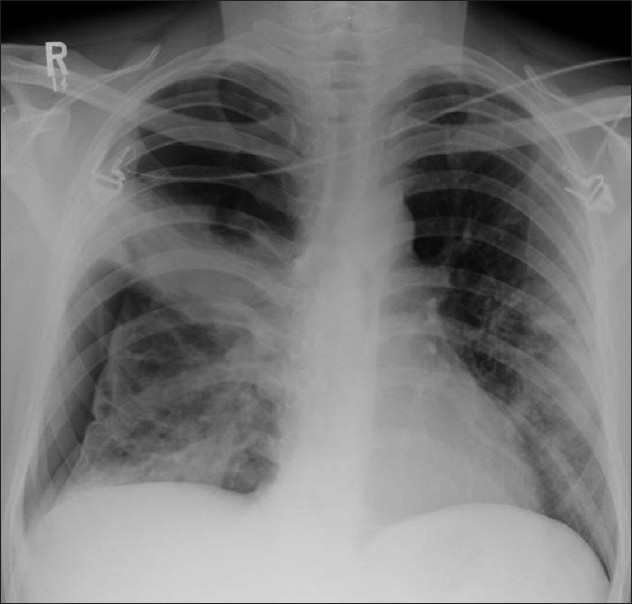
*Pneumocystis carinii* pneumonia. Pneumothoraces are often refractory to conventional chest tube drainage, becoming chronic, requiring pleurodesis or surgical intervention as in this patient

Spontaneous pneumothorax is a well-recognised complication, particularly in patients with cysts [Figure 14]. Pneumothoraces may be bilateral and they have important implications for management and prognosis as patients have a significantly increased mortality rate [Figures 15 and 16]. Pneumothoraces are often refractory to conventional chest tube drainage, becoming chronic and requiring pleurodesis or surgical intervention[40] [Figure 17].

**Figure 18:**
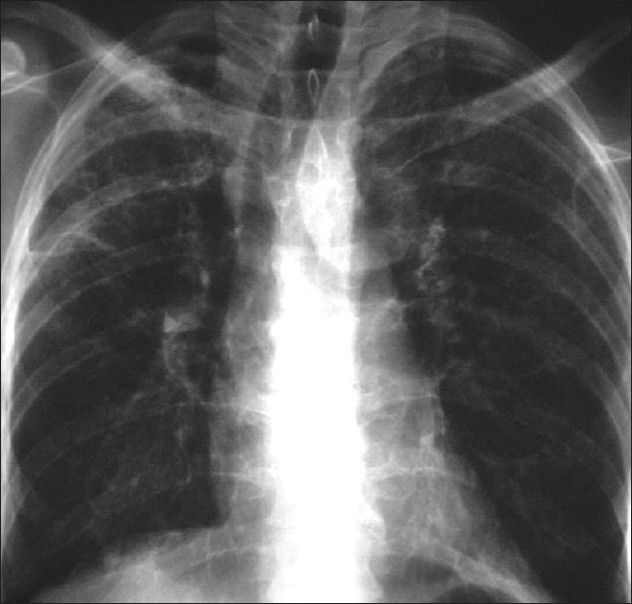
*Pneumocystis carinii* pneumonia. Chest X-ray shows atypical features of upper lobe focal reticulation associated with minor ground-glass appearance

**Figure 19:**
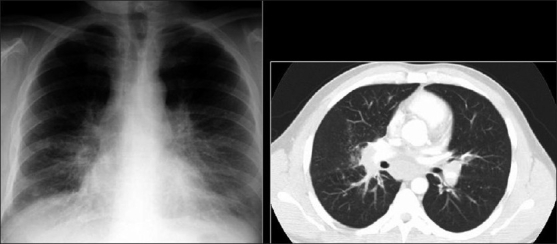
*Pneumocystis carinii* pneumonia. Perihilar haze associated with hilar lymphadenopathy mimicking sarcoidosis

**Figure 20:**
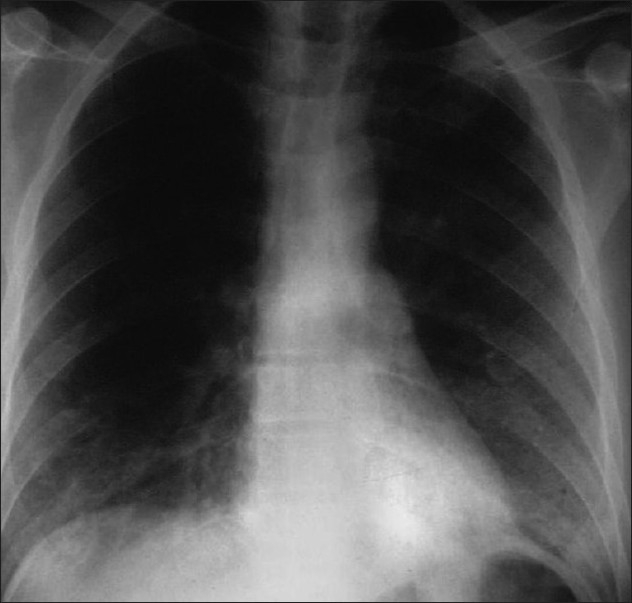
*Pneumocystis carinii* pneumonia atypical features. Chest X-ray shows focal consolidation of the left lung base

**Figure 21:**
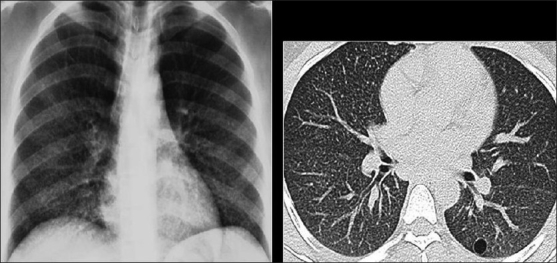
*Pneumocystis carinii* pneumonia atypical features. Chest X-ray shows finely granular/miliary appearance better depicted on high-resolution computed tomography

**Figure 22:**
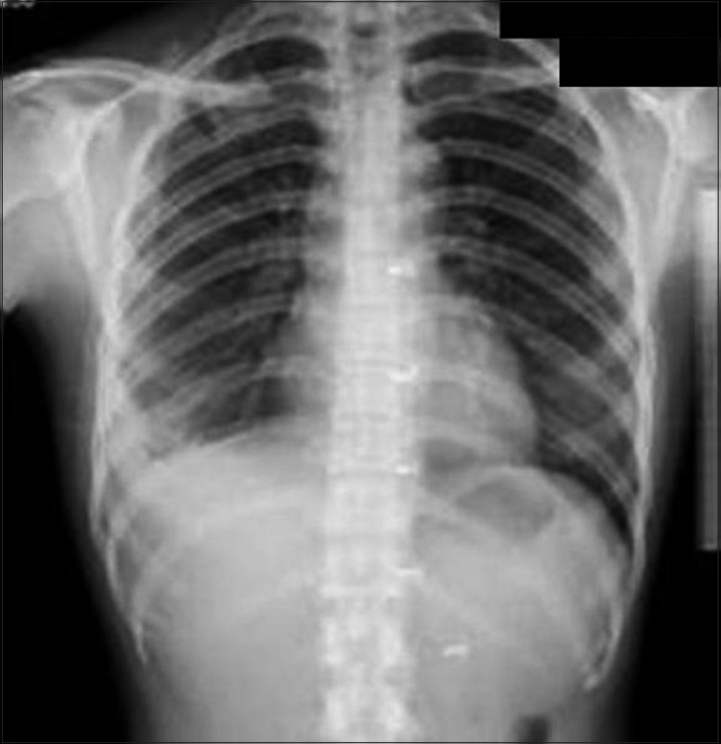
*Pneumocystis carinii* pneumonia atypical features. Chest X-ray shows diffuse, vague, ill-defined lung nodules associated with a right-sided pleural effusion (confirmed by ultrasound)

The CXR may be normal in 10%, although this is uncommon in clinical practice[40,42] Atypical findings are seen in a further 10%, including isolated focal or asymmetrical dense consolidation, airways disease and adenopathy. Effusions are extremely uncommon and should prompt the search for other pathogens. Nodules are another unusual manifestation. Histologically, these represent granulomas. Nodules can vary from miliary to >1 cm in size, and may infrequently undergo cavitation or calcification[35,40] [Figures 18–22].

## Mycobacterial Infections

MTB is the most common pulmonary complication of HIV worldwide.[43] Making the diagnosis may be difficult as patients tend to be anergic. Therefore, imaging has an important role to play in the diagnosis.[43–45]

The radiographic appearance of TB in AIDS differs from that in immune competent hosts, with more diffuse and lower zone disease, miliary disease and adenopathy as well as an increased incidence of a normal CXR.[44,45]

The radiographic appearance generally reflects the CD4 count. When the CD4 level exceeds 200, the appearance is commonly that of typical reactivation MTB in a previously infected immune competent host. Classical primary TB can also be seen in patients without prior exposure. In contrast, patients with CD4 levels below 200 present with a pattern of primary TB, regardless of prior exposure.[43–45]

**Figure 23:**
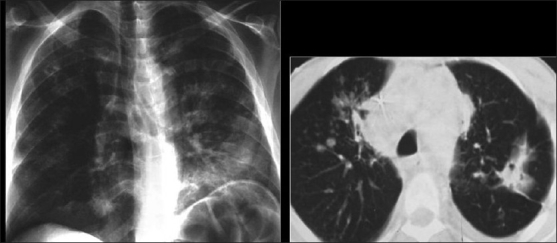
Chest X-ray and computed tomography showing features of reactivation *Mycobacterium tuberculosis* as patchy consolidation, including involvement at unusual sites, e.g. lower lobes as seen here, cavitation, nodularity and adenopathy

**Figure 24:**
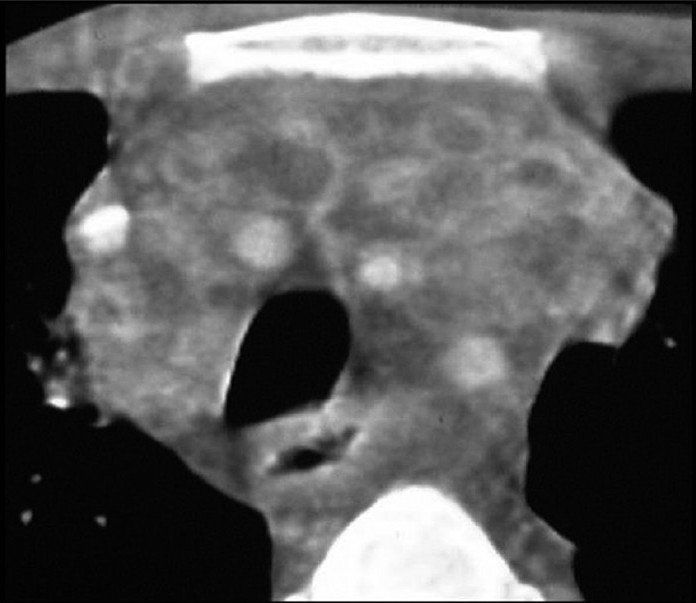
*Mycobacterium tuberculosis* (TB). TB mediastinal lymph nodes are typically markedly enlarged and of low attenuation on computed tomography, often demonstrating rim enhancement following contrast administration, as in this case

Features of reactivation MTB are patchy consolidation, including involvement at unusual sites, e.g. lower lobes, cavitation, nodularity, effusions and adenopathy [Figure 23]. TB lymph nodes are typically markedly enlarged and of low attenuation on CT, often demonstrating rim enhancement following contrast administration[43–45] [Figure 24].

**Figure 25:**
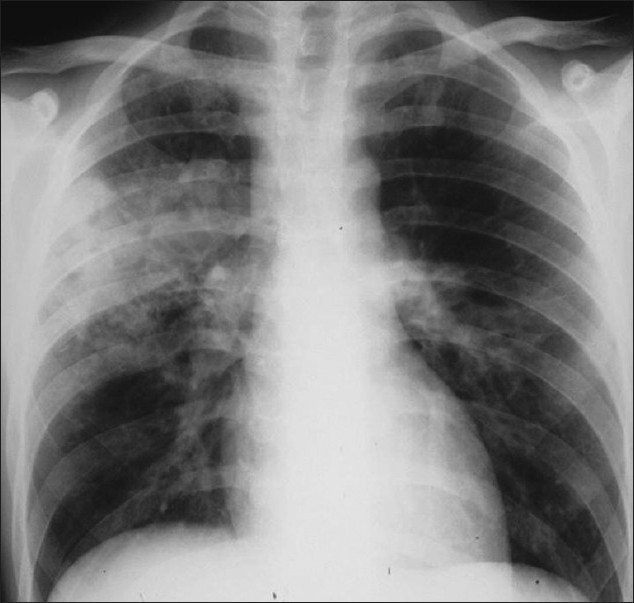
“Primary” *Mycobacterium tuberculosis*. Chest X-ray shows right upper lobe and left midzone consolidation and adenopathy. Note lack of cavitation in this patient with a low CD4 count

**Figure 26:**
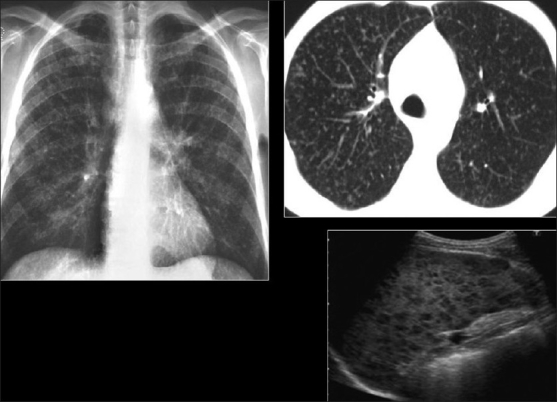
“Primary” *Mycobacterium tuberculosis (MTB)*. Chest X-ray and high-resolution computed tomography show diffuse randomly distributed miliary nodules. Ultrasound of the spleen of the same patient shows splenomegaly and multiple hypoechoic nodules due to MTB granulomas

**Figure 27:**
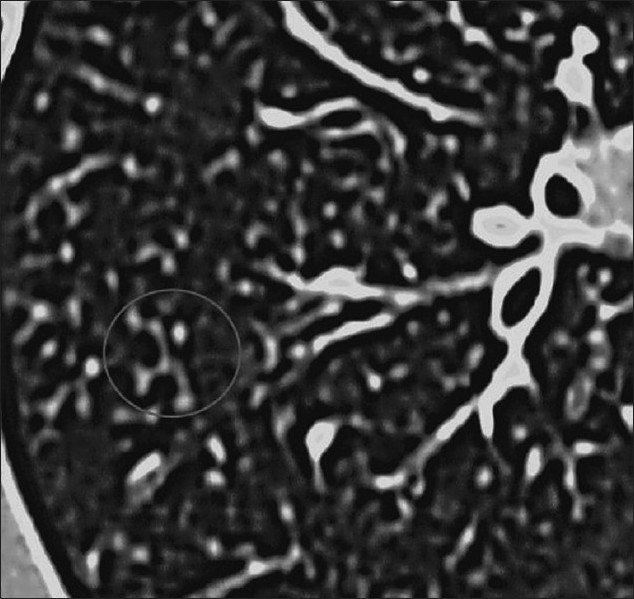
*Mycobacterium tuberculosis*. High-resolution computed tomography shows a tree-in-bud appearance

“Primary” MTB manifests as focal unilateral, often lower lobe consolidation and adenopathy[46] [Figure 25]. Cavitation is less common at lower CD4 counts. Patients in this range also have an increased incidence of miliary MTB, with diffuse, randomly distributed nodules on CT [Figure 26]. Approximately 15% of the patients have normal CXRs.[44] This particularly occurs when disease is isolated to the airways. Alternatively, the CXR may demonstrate an asymmetric reticulonodular pattern. However, the HRCT is invariably abnormal, typically demonstrating adenopathy and the “tree-in-bud” pattern, similar to bacterial bronchiolitis[45] [Figure 27]. However, this is usually asymmetric and involves upper as well as lower lobes in distinction to bacterial infection.

Serial CXRs are useful for monitoring response to treatment.[43–45] A paradoxical response to treatment with transient worsening of appearances within 1 month of commencing anti-TB therapy is a recognized phenomenon.[47] This is more frequent in AIDS patients receiving HAART due to increased inflammatory responses secondary to improved host immunity. Severe worsening may occur, particularly in advanced immune suppression.[47]

Although the time frame and clinical context of immune restoration syndrome are characteristic, radiographic findings are nonspecific, and this should only be diagnosed when other causes, such a poor compliance with treatment, drug resistance, drug reaction and concurrent disease, have been excluded. The latter, in particular, because almost 30% of the patients have been shown to develop new HIV-related lung disease during the first 3–6 months of TB treatment, most commonly PCP.[47]

## Nontuberculous/Atypical Mycobacterium (NTMB)

**Figure 28:**
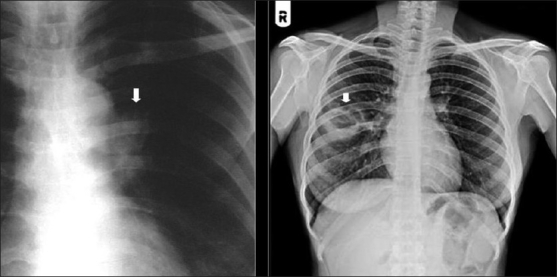
Nontuberculous/atypical Mycobacterium (NTMB). The chest X-ray (CXR) abnormalities resemble *Mycobacterium tuberculosis*, with focal consolidation CXR (left), diffuse patchy infiltrates and cavities (right). The features are, however, nonspecific and the diagnosis is often delayed

**Figure 29:**
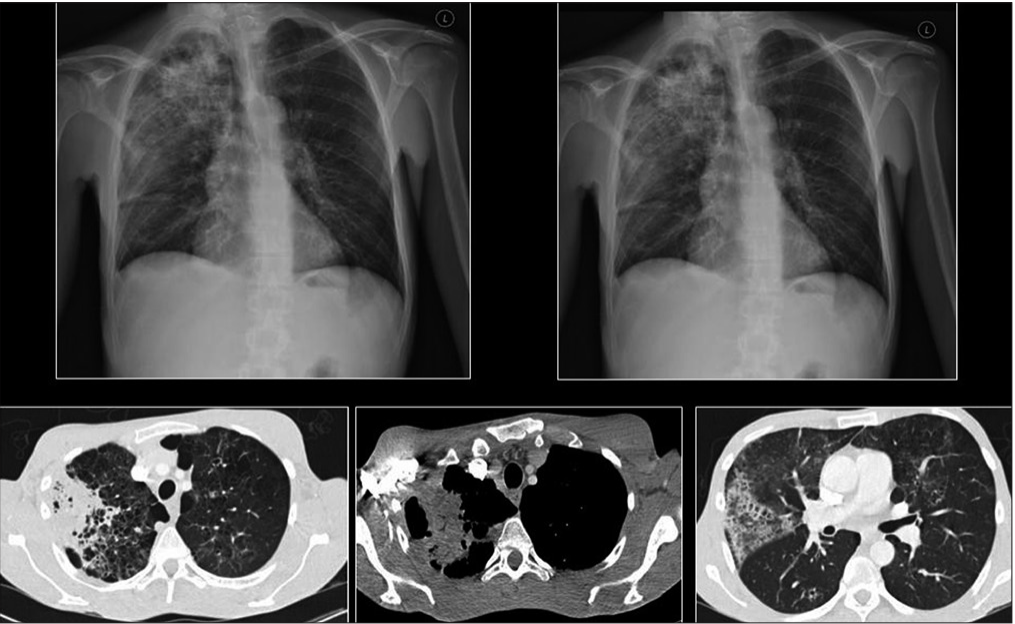
*Mycobacterium xenopi* in a human immunodeficiency virus, 36-year old, male patient with a CD4 count of 80 with four positive sputum samples and a bronchoalveolar lavage for acid-fast bacilli. The chest X-ray and computed tomography scans show cavitating consolidation, loss of volume, traction bronchiectasis and ground-glass appearances in the right apical region superimposed on bullous disease of the lungs. There is no associated lymphadenopathy

**Figure 30:**
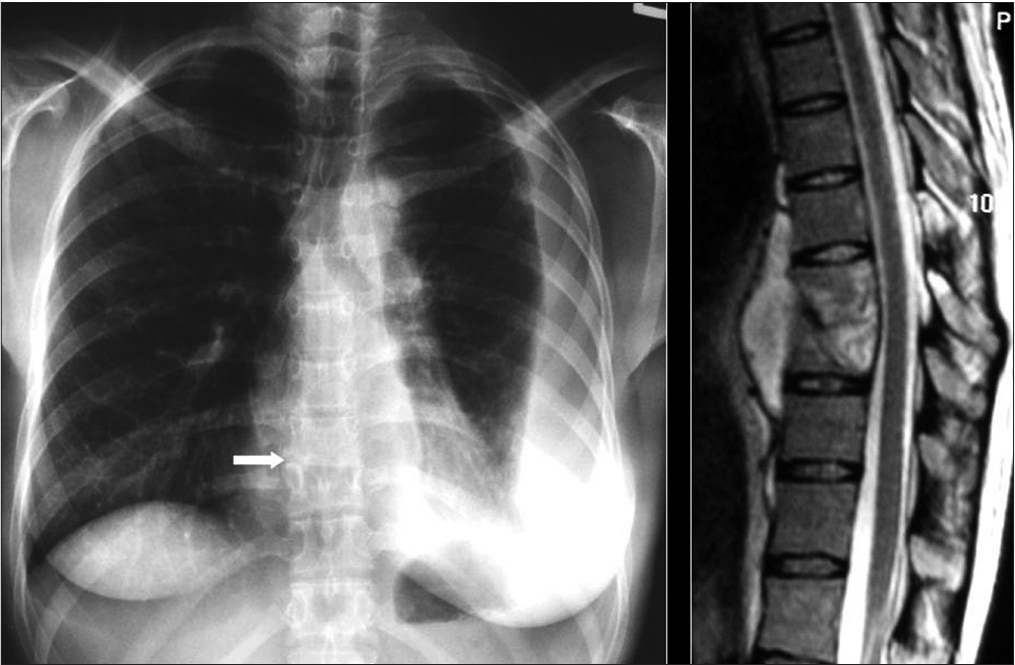
A 26-year-old human immunodeficiency virus-positive female presented with shortness of breath. The chest X-ray shows a large left-sided pleural effusion and loss of height and erosion of the articular plates between the 9^th^ and 10^th^ vertebral bodies associated with soft tissue swelling. A sagittal T2-weigted magnetic resonance scan of the dorsal spine shows complete obliteration of the disc between the 9^th^ and 10^th^ dorsal vertebral bodies associated with fluid collection anterior to the spine representing pus. Acid-fast bacilli were identified in the aspirated pus

MAC is the most common atypical mycobacterial infection.[35,47,48] It usually causes disseminated disease with pulmonary involvement in only 5%.[35,47,48] Clinically significant pneumonia is uncommon and the CXR is often normal, even if sputum cultures are positive.[35,47,48] Radiographic abnormalities resemble MTB, with focal consolidation, diffuse patchy infiltrates with upper lobe predominance, nodules and cavities [Figures 28–30]. The features are, however, nonspecific and the diagnosis is often delayed.[35,47,48]

## Cryptococcus

**Figure 31:**
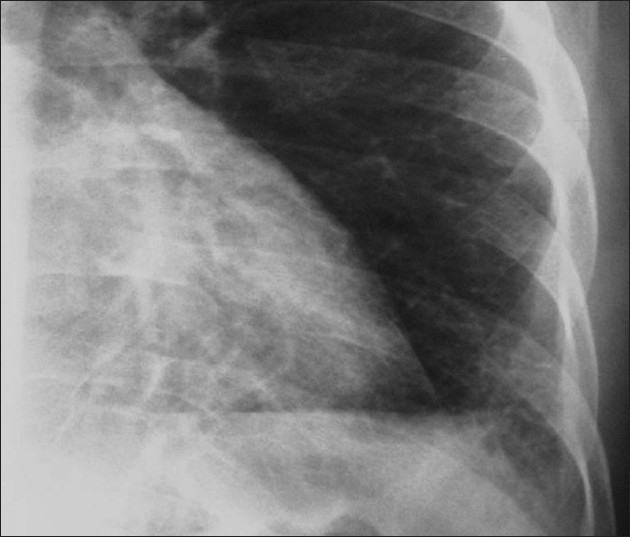
Cryptococcosis. Imaging findings are varied and nonspecific. Reticular chest X-ray or reticulonodular infiltrates are the most common pattern as in this case where a reticulonodular infiltrate involved the left costophrenic angle

**Figure 32:**
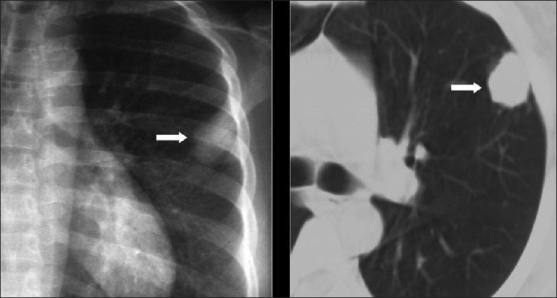
Cryptococcosis. Chest X-ray and computed tomography show a solitary pulmonary nodule. The diagnosis of Cryptococcosis was confirmed on biopsy

**Figure 33:**
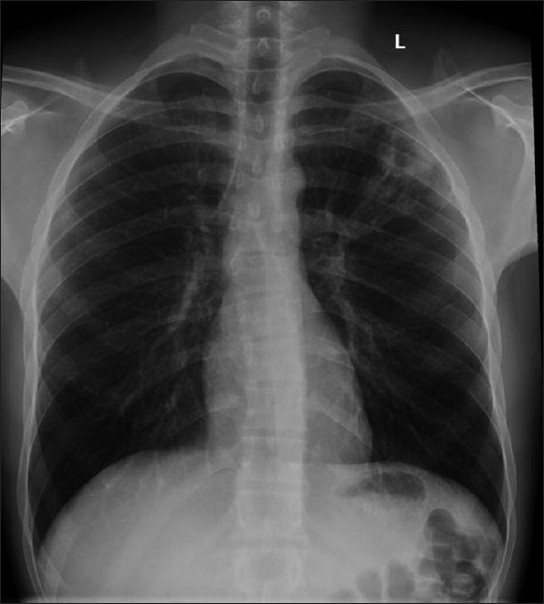
Cryptococcosis. Chest X-ray shows a less-frequently seen cavitation due to Cryptococcosis in an acquired immunodeficiency syndrome patient

**Figure 34:**
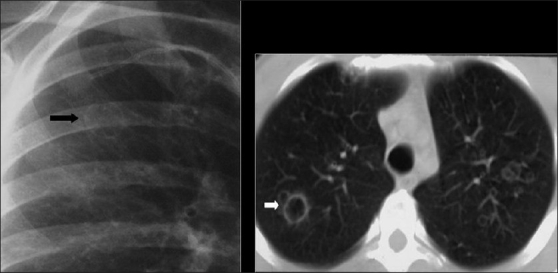
Cryptococcosis. Chest X-ray (L) shows a thin-walled cavity (arrow) associated with patchy consolidation. Computed tomography section at the level of the lower trachea (R) shows multiple cavities of varying sizes associated with subtle ground-glass opacification

**Figure 35:**
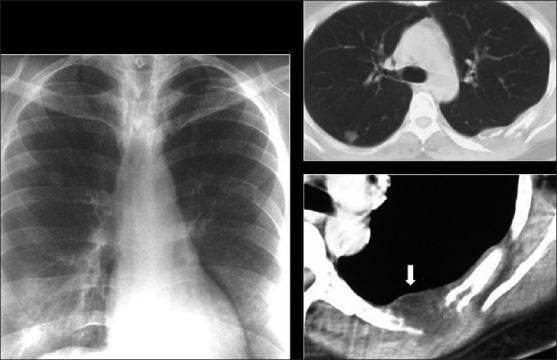
Cryptococcosis. Chest X-ray and computed tomography show lessfrequent manifestations of lung nodules and a chest wall abscess (arrow)

Cryptococcus is the fourth most common opportunistic infection in AIDS.[35,48] The lungs are the portal of entry and the most common site of infection although, the common clinical presentation is with meningitis, pneumonia occurring in only approximately 30%.[35,48] Imaging findings are varied and nonspecific. Reticular or reticulonodular infiltrates are the most common pattern[35,49] [Figure 31]. Solitary or multiple nodules [Figure 32], often up to 4 cm in diameter, are seen in around 30%. Biopsy is usually required for diagnosis. Cavitation occurs less frequently in AIDS-related disease compared to immune competent hosts, usually appearing early in the course of the illness, when the level of immune suppression is mild[35,49,50] [Figures 33 and 34]. Less-frequent manifestations include adenopathy, effusions, consolidation, miliary nodularity and ground-glass opacification and chest wall abscesses[35,49] [Figure 35].

### Aspergillus

**Figure 36:**
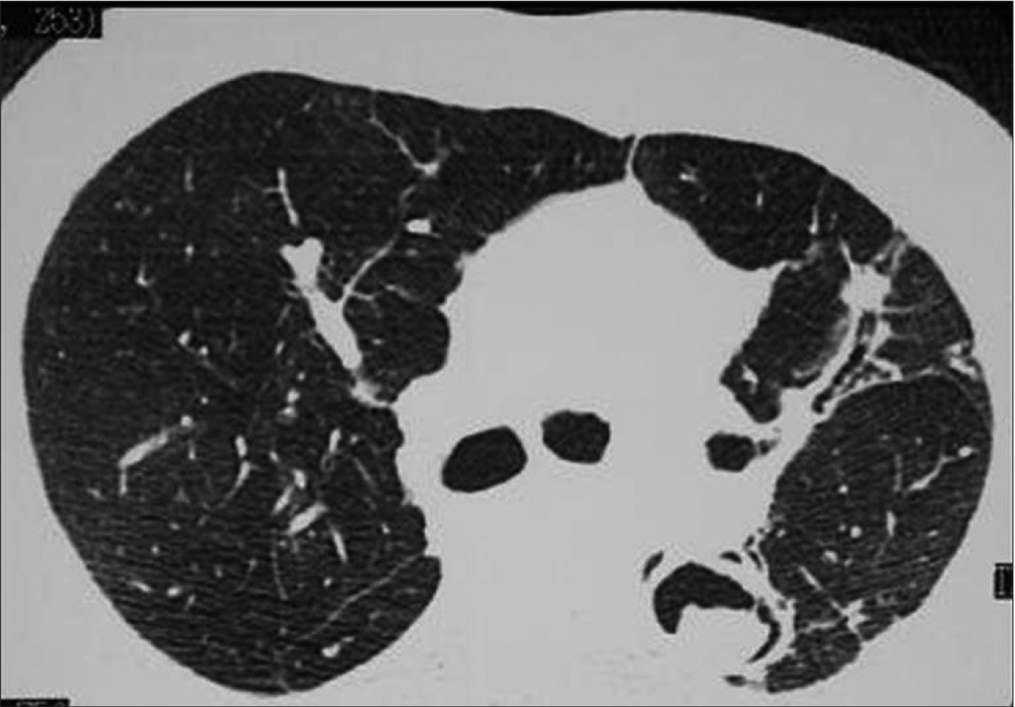
Aspergillosis. Mycetomas are the least common, but can complicate cavitary *Mycobacterium tuberculosis* or *Pneumocystis carinii* pneumonia. The computed tomography here represents a mycetoma in a tuberculous cavity. Note the traction bronchiectasis and loss of volume in the left upper zone

**Figure 37:**
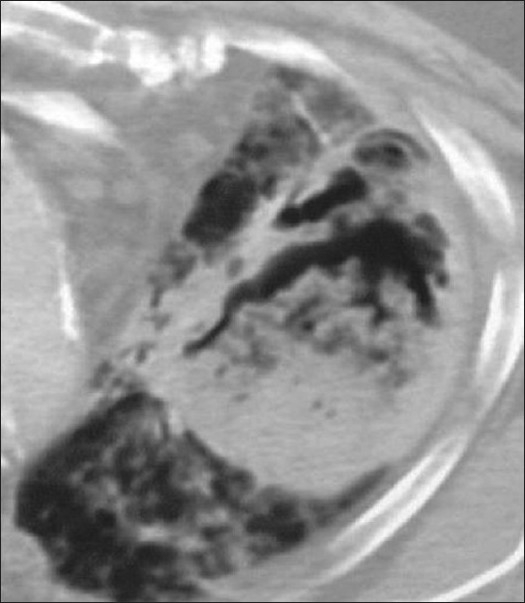
Aspergillosis. Angioinvasive disease chest X-ray is the most common, manifesting as thick-walled cavitary lesions predominating in the upper lobes, with air-crescents surrounding areas of desquamated infarcted lung. Here, we see all the described features on computed tomography

**Figure 38:**
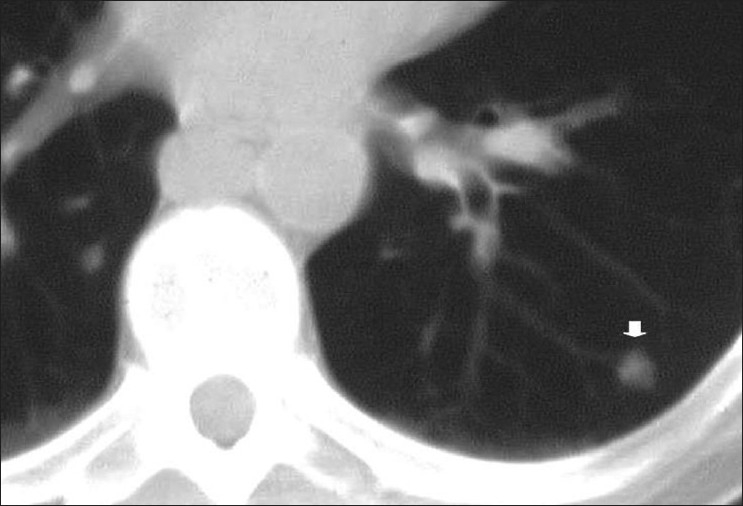
Aspergillosis. Less-common patterns include computed tomography nodules (arrow) with a peripheral halo of ground-glass attenuation (not shown)

**Figure 39:**
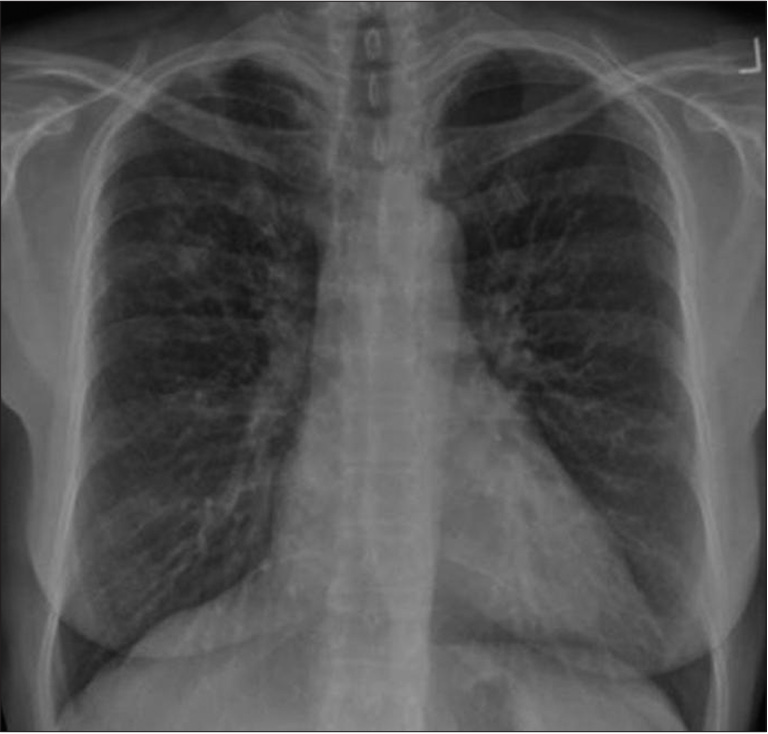
Aspergillosis. ABPA chest X-ray (CXR), manifesting as bilateral consolidation, mostly lower lobe, bronchiectasis and airway impaction or “finger in glove.” The consolidation in the CXR shown is much more diffuse

**Figure 40:**
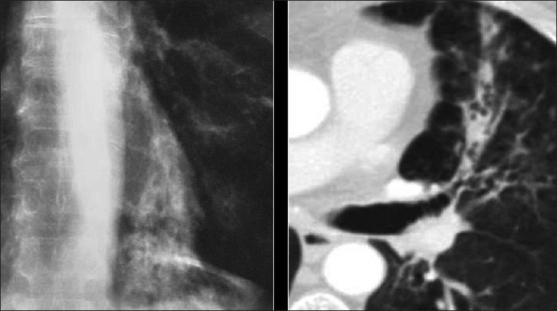
Aspergillosis. ABPA a chest X-ray/computed tomography on the same patient shows bilateral lower lobe consolidation, bronchiectasis and airway impaction or “finger in glove” appearance

Aspergillosis occurs almost exclusively in association with neutropoenia, usually secondary to drug therapy.[35,50] The incidence is reported to be around 1%, but is thought to be increasing due to prolonged survival at advanced levels of immune suppression.[50] All forms have been described, often coexisting in the same patient. Mycetomas are the least common, but can complicate cavitary MTB or PCP[35,50] [Figure 36]. Clinical progression to invasive Aspergillosis is reported to occur in 50%, despite antifungal therapy, whereas this is not a feature of the disease in immune competent hosts. Angioinvasive disease is most common, manifest as thick-walled cavitary lesions predominating in the upper lobes, with air-crescents surrounding areas of desquamated infarcted lung[35,50] [Figure 37] Less-common patterns include nodules with a peripheral halo of ground-glass attenuation and isolated airway disease [Figure 38] or Allergic bronchopulmonary Aspergillosis (ABPA), manifesting as bilateral lower lobe consolidation, bronchiectasis and airway impaction or “finger in glove”[35,50] [Figures 39 and 40].

## CMV

Ninety percent of HIV-positive patients are thought to carry latent CMV infection, although it is not considered a significant pulmonary pathogen in the majority.[35] CMV pneumonitis is thought to be increasing in incidence likely secondary to enhanced life expectancy and changing demographics with increased heterosexual HIV transmission, which is associated with CMV infection.[35]

**Figure 41:**
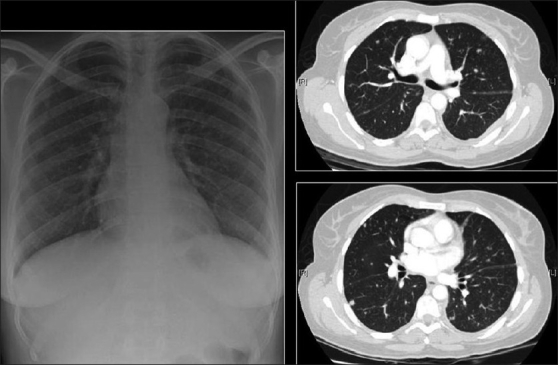
Cytomegalovirus (CMV). Human immunodeficiency virus patient with a CD4 count of 40 presented with fever and dry cough. The chest X-ray shows ill defined diffuse small nodules. High-resolution computed tomography scans confirm scattered bilateral centrilobular nodule-associated interlobular septal thickening. Imaging appearances of CMV pneumonia are nonspecific and may mimic other opportunistic infections

**Figure 42:**
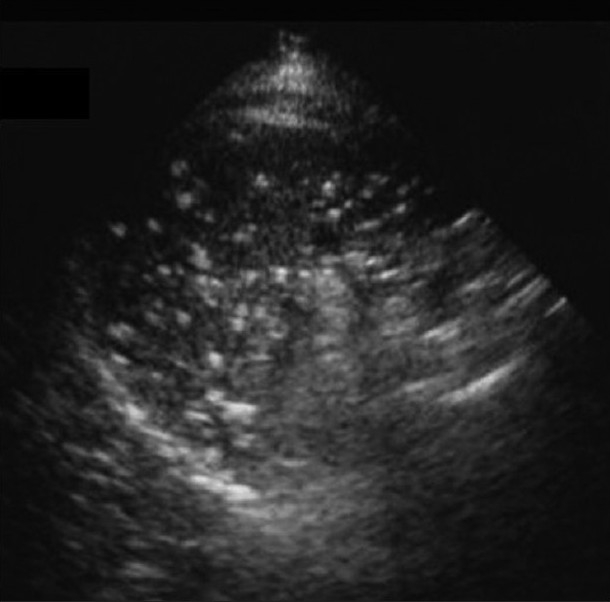
Ultrasound of the spleen on the same patient as in Figure 41 showing multiple microabscesses due to systemic cytomegalovirus infection

The spectrum of radiographic findings is varied and overlaps other AIDS-related diseases, most notably PCP.[35] Features include ground-glass opacification, nodules (varying from miliary to 3 cm), perihilar and lower zone interstitial infiltrates and effusions. When ground-glass opacification is associated with nodularity and an effusion, CMV should be considered over PCP, especially in patients with CD4 counts below 50.[51] Small airway disease may be the sole manifestation of infection[51] [Figures 41 and 42].

## KS

KS is by far the most common AIDS-related malignancy.[35] The male:female ratio is 50:1.[35] KS Herpes virus or Human Herpes virus 8 has been identified as the probable cause, likely in conjunction with cofactors.[35–52] Pulmonary involvement occurs in up to 50% and is almost always preceded by cutaneous or visceral disease, although the latter may not always be recognized.[53]

**Figure 43:**
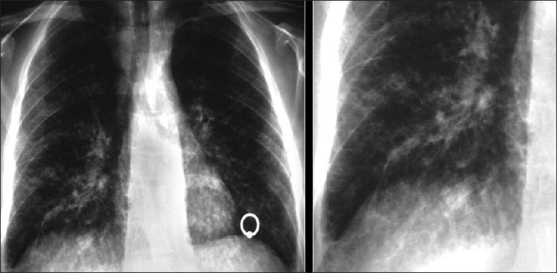
Kaposi’s sarcoma is the most common acquired immunodeficiency syndrome-related malignancy. The chest X-ray shows bilateral perihilar/lower zone reticulonodular infiltrates

**Figure 44:**
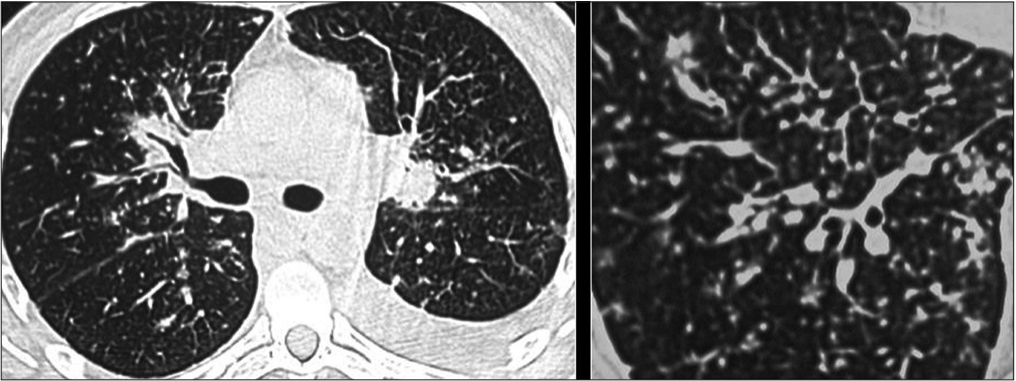
Kaposi’s sarcoma. High-resolution computed tomography shows thickening of the bronchovascular bundles reflecting bronchocentric disease. Interlobular septal thickening due to lymphatic obstruction because of tumor invasion is also seen

Bilateral, perihilar and lower-zone reticulonodular infiltrates are characteristic [Figure 43]. Septal lines may also be visible. “Flame-shaped” nodules or masses are another characteristic finding classically associated with a halo of ground-glass attenuation on CT[35,42] [Figure 44]. Although adenopathy has been described, this is rarely significant, and while endobronchial lesions are identified in up to 75% of the patients bronchoscopically, they are less commonly appreciated on CT.[35,42]

**Figure 45:**
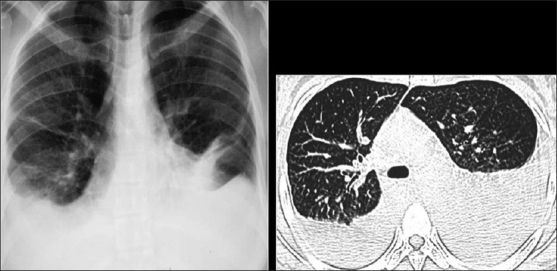
Kaposi sarcoma. Pleural effusions chest X-ray/computed tomography are common. They may be unilateral or bilateral and may be large and characteristically hemorrhagic on aspiration

Thickening of bronchovascular bundles reflecting the bronchocentric distribution of disease is demonstrated on HRCT. Interlobular septal thickening due to lymphatic obstruction and tumor invasion is also seen[35,42,54] [Figure 44]. Pleural effusions are common, present in around 30%. They may be unilateral or bilateral and may be large and characteristically hemorrhagic on aspiration[35,42,54] [Figure 45].

## Lymphoma

AIDS-related lymphoma (ARL) is the second most common malignancy.[52,55] The incidence is increasing, possibly due to the longer life expectancy coupled with the longer latency period required for the development of neoplasms.[56]

Non-Hodgkin’s Lymphoma (NHL) accounts for 90% and the majority of cases are associated with Epstein-Barr virus. NHL is typically extranodal and usually disseminated at the time of diagnosis.[52] Thoracic involvement is reported in up to 40%, although subclinical involvement is probably higher.[52,57]

**Figure 46:**
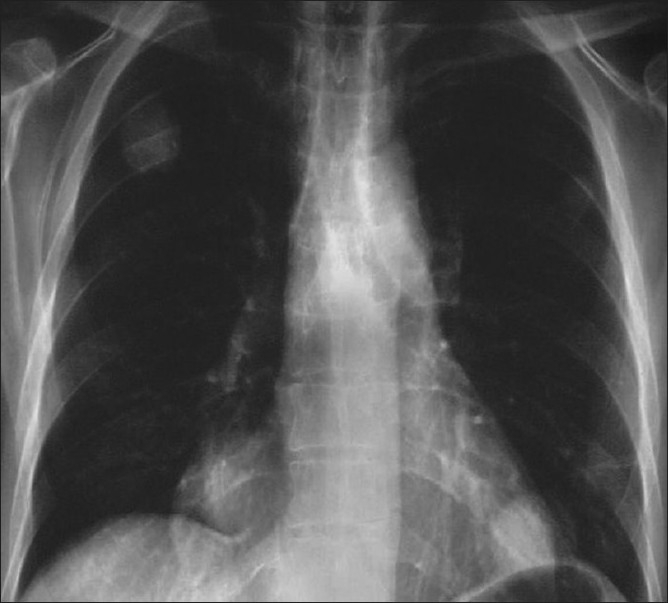
Lymphoma. Chest X-ray (CXR) on a human immunodeficiency virus patient that presented with multiple lung masses, which grew rapidly mimicking infection. Note that there is no associated lymphadenopathy. Well-defined solitary or multiple parenchymal nodules CXR are common. A percutaneous biopsy revealed a non-Hodgkin’s lymphoma

**Figure 47:**
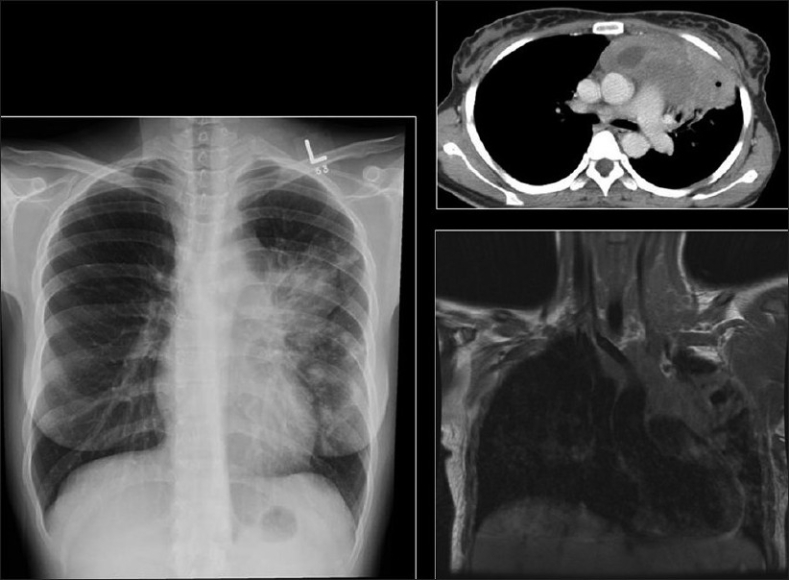
NHL in a 23-year-old human immunodeficiency virus female. The chest radiograph shows multiple well-defined lung nodules within the left lung associated with mediastinal lymphadenopathy. Lymphadenopathy is a less common feature in acquired immunodeficiency disease-related NHL and nodes are rarely significant according to size criteria unlike as in the case shown here, where there is significant lymphadenopathy as confirmed by computed tomography (right upper frame). Magnetic resonance imaging is the imaging of choice to detect vascular encasement

Well-defined solitary or multiple parenchymal nodules are common [Figure 46]. These are frequently large and often demonstrate a very short doubling time of between 4 and 6 weeks, mimicking infection. Unlike KS, they are often peripheral and while air-bronchograms may be seen, cavitation is unusual[55,57] [Figure 47].

Lymphadenopathy is a less common feature in AIDS-related NHL, and nodes are rarely significant according to size criteria. Effusions are common, may be unilateral or bilateral and are usually moderate to large in size.[58]

A rare form of ARL, called body cavity lymphoma, has been described, usually affecting homosexual males with advanced AIDS, manifest as pleural, pericardial or peritoneal effusions in the absence of any soft tissue tumor.[59–61]

## Lung Carcinoma

**Figure 48:**
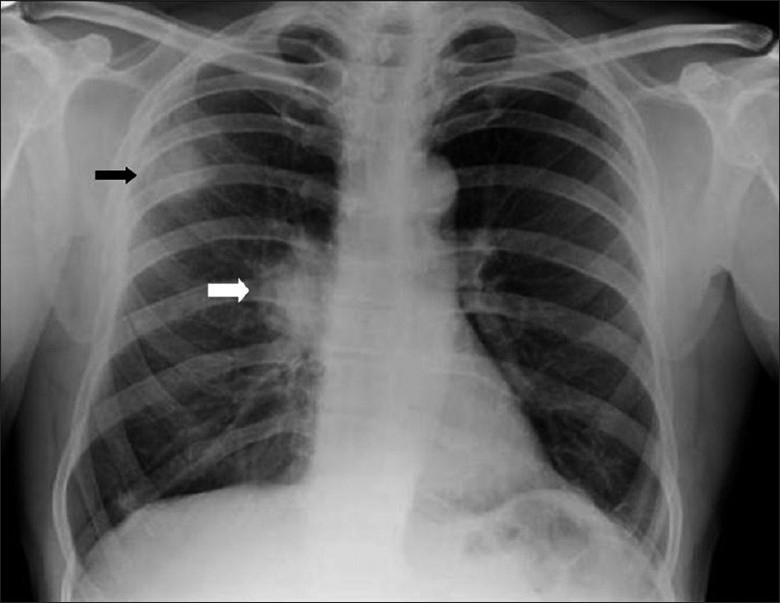
Bronchogenic carcinoma. Radiographic appearances are similar to ordinary lung cancer, except that lesions tend to be more peripheral and in the upper lobes as in this case (black arrow). Note the right hilar lymphadenopathy (white arrow)

There is conflicting data regarding the incidence of lung carcinoma in AIDS and, to date, there is no convincing evidence showing a significant rise.[56,58,62] It occurs in smokers, although important clinical differences have been noted compared with the general population.[58] There is a striking male preponderance and patients often present at a younger age and at a later stage.[58,62] There is no correlation between stage of disease and CD4 count. Tumors are frequently poorly differentiated or predominantly adenocarcinomas.[58,63] Radiographic appearances are similar to ordinary lung cancer, except that lesions tend to be more peripheral, with over 90% in the upper lobes.[58,64] Extensive pleural disease as the sole radiographic manifestation of lung cancer has also been reported[58,64] [Figure 48].

## Lymphoproliferative Disorders

**Figure 49:**
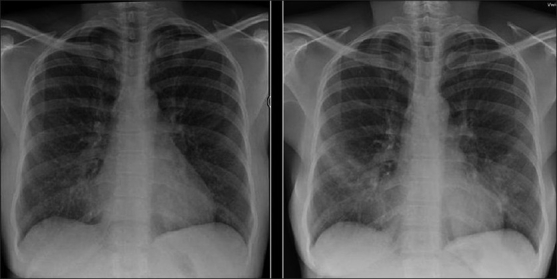
Lymphocytic interstitial pneumonitis in a human immunodeficiency virus patient. Chest X-ray showing bilateral reticulonodular interstitial infiltrates. Diagnosis was confirmed by a transbronchial biopsy

**Figure 50:**
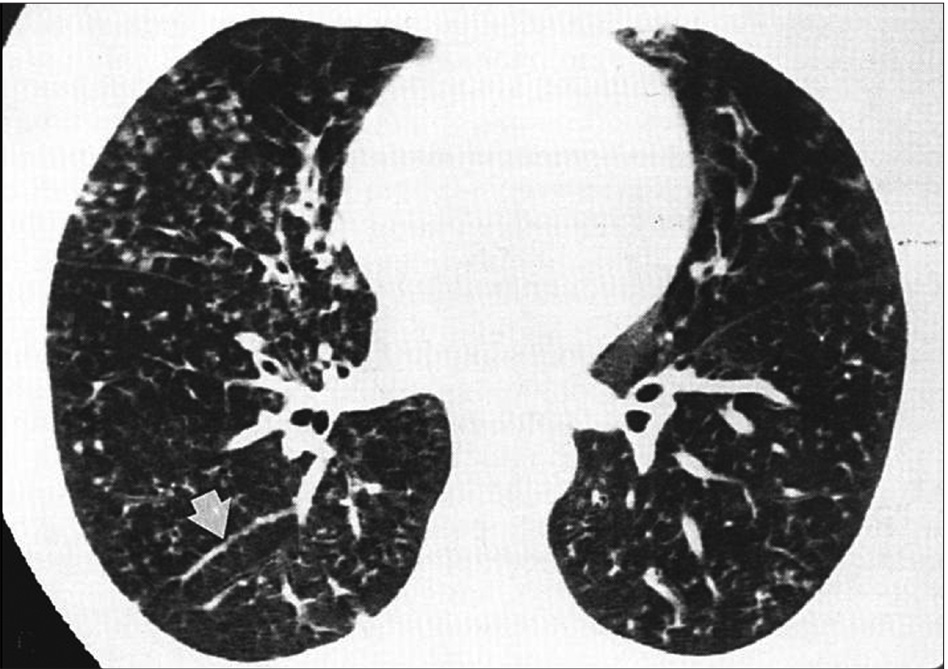
Lymphocytic interstitial pneumonitis in a human immunodeficiency virus patient. High-resolution computed tomography shows smooth and nodular thickening of bronchovascular bundles, centrilobular and subpleural nodularity, ground-glass opacification and interlobular septal thickening. Cysts and mediastinal adenopathy may also be seen (not shown here)

A spectrum of polyclonal lymphoproliferative disorders affects the lungs of HIV-positive patients, the most common being lymphocytic interstitial pneumonitis.[35,65] This is thought to represent lymphoid hyperplasia in response to chronic antigenic stimulation by the AIDS virus.[35,65] It is far more common in children than in adults.[66] The diagnosis is made on transbronchial biopsy. On CT, there is smooth and nodular thickening of bronchovascular bundles, centrilobular and subpleural nodularity, ground-glass opacification and interlobular septal thickening.[65,67,68] Cysts and mediastinal adenopathy may also be seen[68] [Figures 49 and 50].

## Bronchiolitis Obliterans

**Figure 51:**
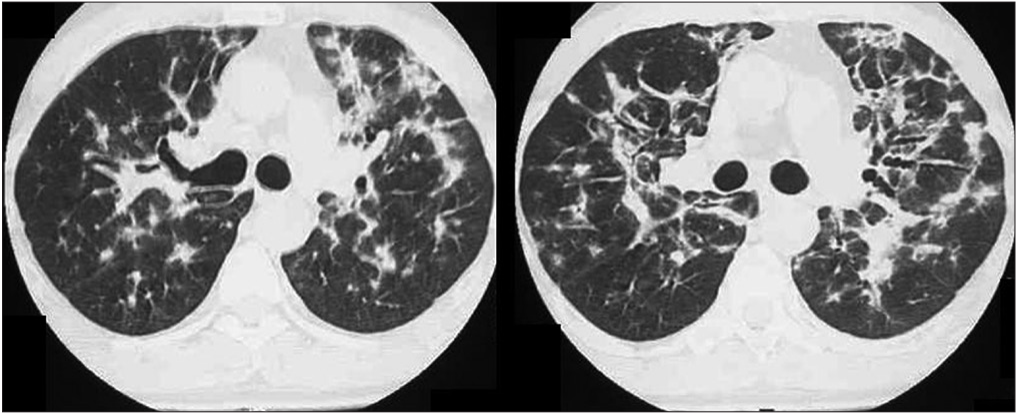
Bronchiolitis obliterans with or without organizing pneumonia in the absence of infection can be a feature of acquired immunodeficiency syndrome (AIDS). This is an infrequent imaging diagnosis, although focal air trapping on expiratory computed tomography, consistent with bronchiolitis obliterans, has been demonstrated in two-thirds of human immunodeficiency virus-positive patients without AIDS, the severity increasing with the duration of infection

Bronchiolitis obliterans, with or without organizing pneumonia in the absence of infection, can be a feature of AIDS.[69–72] This is an infrequent imaging diagnosis, although focal air trapping on expiratory CT, consistent with bronchiolitis obliterans, has been demonstrated in two-thirds of HIV-positive patients without AIDS, the severity increasing with the duration of infection.[73] However, because definitive diagnosis requires lung biopsy, the true incidence of disease is unknown and, in clinical practice, the diagnosis is usually one of exclusion. The radiographic appearance is as for non-AIDS[73] [Figure 51].

## Emphysema

**Figure 52:**
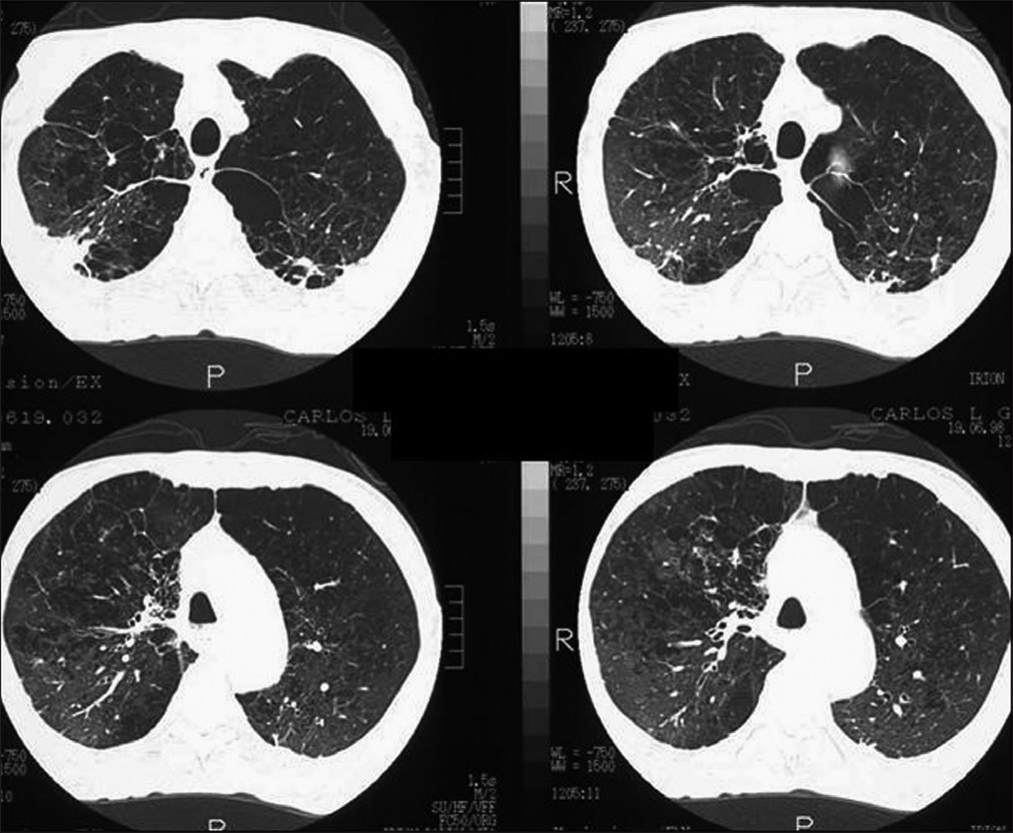
High-resolution computed tomography of a 46-year-old man with acquired immunodeficiency syndrome-related emphysema. This patient was also a smoker and experimented with cocaine and marijuana. A biopsy revealed destruction of the lung parenchyma distal to the terminal bronchioles accompanied by various degrees of inflammation

Recently, work suggests that there may be a higher incidence of pulmonary emphysema among HIV-positive smokers.[71] The probable cause may lie in the susceptibility of HIV patients to recurrent chest infections[71] [Figure 52].

## Cardiovascular Complications

**Figure 53:**
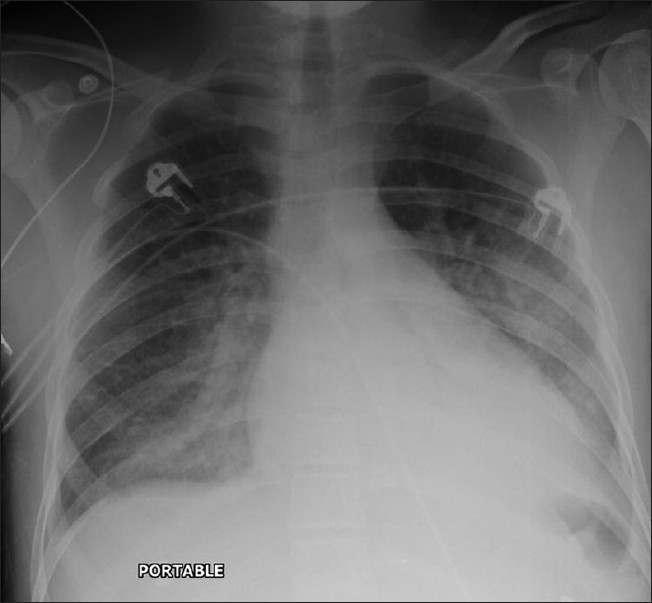
Anterioposterior chest radiograph on a 43-year-old man treated with highly active antiretroviral therapy for over 8 years who presented with increasing shortness of breath over the past 3 months that had suddenly worsened, prompting hospital admission. The chest X-ray shows features of pulmonary edema. Subsequent investigations revealed congestive cardiomyopathy

**Figure 54:**
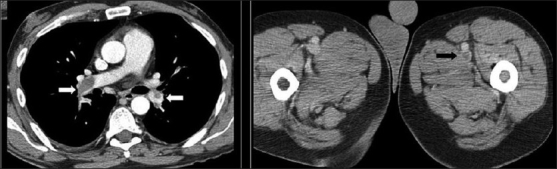
Thromboembolism. Computed tomography (CT) images from a 38-year old human immunodeficiency virus-positive man with a CD count of 400 presented with acute tightness in the chest and low O_2_ saturation. The CT scans show pulmonary emboli within the lower lobe pulmonary arteries on both sides (white arrows). Note the thrombus in the left femoral artery (black arrow)

**Figure 55:**
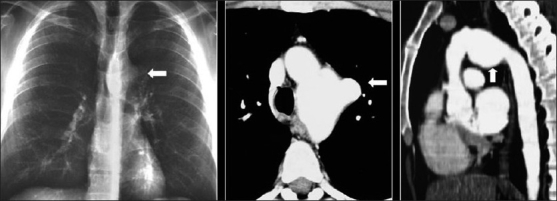
This human immunodeficiency virus patient suffered from gram negative septicemia, which was successfully treated. However, routine physical examination revealed an audible bruit on thoracic auscultation. The chest X-ray shows a prominent hump over the proximal descending aorta due to mycotic aortic aneurysm. The axial computed tomography (CT) and coronal CT reconstruction elegantly demonstrate the abnormality

**Figure 56:**
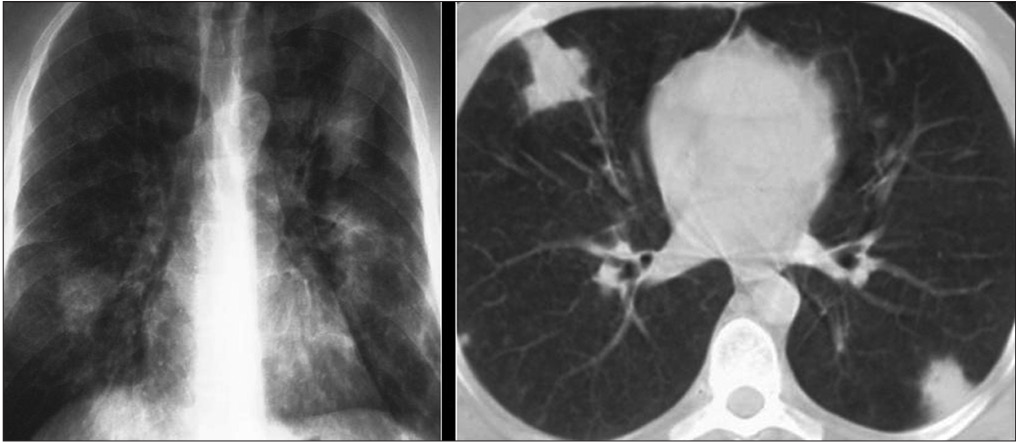
This hemophilic patient was infected with human immunodeficiency virus (HIV) from factor 8 before HIV testing became compulsory. The images show pulmonary hemorrhage on the background of *Pneumocystis carinii* pneumonia

Better prophylaxis for opportunistic infections and the development of HAART has had a significant impact against HIV on viral load, CD4^+^cell count and HIV-related mortality in HIV patients.[7] With longer survival, cardiovascular complications are becoming manifest in these patients. Cardiovascular complications of HIV include cardiomyopathy and pulmonary arterial hypertension [Figure 53]. A higher incidence of atherosclerosis has been reported possibly secondary to protease inhibitor therapies.[74] A recent study lists HIV as a risk factor for deep venous thrombosis and pulmonary embolism[75] [Figure 54]. Finally, a couple of cases do not fit into the categories already described, including mediastinal vascular complications [Figure 55]. Lastly, it would be difficult to predict the biopsy-proven diagnosis in patients with ill-defined centrilobular nodules and confluent mass-like areas of consolidation without knowing that they were hemophiliac and had AIDS [Figure 56].

## Radiographic Patterns

Limitations reflecting overlapping appearances, atypical manifestations and coexisting disease contribute to a relative lack of specificity of imaging. However, certain basic radiographic patterns can be recognized, which should raise the suspicion of certain underlying disease processes.

## Nodules

Pulmonary nodules are a relatively common manifestation of AIDS-related pulmonary disease. The authors of two recent studies categorized nodules according to size and distribution and showed that nodules smaller than 1 cm, especially those with a random or centrilobular distribution, are more likely to be due to infection (infections being the most common overall cause of multiple nodules), whereas nodules larger than 1 cm are more likely neoplastic. Miliary nodularity is typically due to fungal infection or TB, although, rarely, it may be seen with PCP. KS tends to be peribronchovascular as opposed to lymphoma and lung cancer, which are more peripheral.[76]

## Cavities

Another study showed that cavitating lesions were universally infective in etiology. Eighty-five percent were polymicrobial, the majority involving bacteria (predominantly due to mixed infections often involving Staphylococcus and Pseudomonas). The remainder included MTB, PCP, fungi and CMV.[77–81]

## Adenopathy

Adenopathy is also most commonly due to infection. Although isolated lymphadenopathy can be seen in association with Cryptococcus, TB is by far the most common cause, accounting for about 85% of the cases. Both may be associated with low attenuation adenopathy. Lung cancer should also be included in the differential diagnosis. Calcified adenopathy has been described in PCP. KS may be associated with hyperattenuating lymphadenopathy due to vascular enhancement. This has been shown to have a 70% PPV.[82]

## Focal Consolidation

This is usually due to infection. A report in clinical radiology identified bacterial pneumonia as the most common cause of focal consolidation in AIDS, but showed that Pneumocystis was the most common individual pathogen to cause the appearance, usually as an upper lobe infiltrate. Consolidative PCP, however, rarely has a segmental pattern, helping in distinguishing it from bacterial infection. MTB, MAC, fungi (especially Cryptococcus), mixed infections and, rarely, neoplasms such as lymphoma and KS may also be responsible.[67,83,84]

## Pleural Effusion

The majority of effusions in AIDS are small, occurring with equal incidence in both infection and malignancy. The three main causes are bacterial infection, MTB and KS. Infective causes tend to be associated with unilateral effusions whereas those in KS tend to be bilateral. Non-AIDS-related conditions such as pulmonary embolism (PE) and organ failure should also be considered.[57,60,85–87]

## Conclusion

Despite the development of effective antiviral therapies and better prophylaxis of opportunistic infections, pulmonary complications of HIV/AIDS remain an important cause of morbidity and mortality. We have provided a pictorial assay of the lung afflictions seen in HIV/AIDS and have discussed the interpretation of imaging studies based on pattern recognition read in conjunction with demographic, clinical and laboratory data. Presently, a CXR remains the mainstay of thoracic imaging in HIV-infected patients, but CT/HRCT plays an increasingly important complementary role in establishing an accurate diagnosis when CXR findings are equivocal or nonspecific.
